# Assessment of Pulmonary Function in Children with Juvenile Idiopathic Arthritis: A Cross-Sectional Study

**DOI:** 10.3390/children12030309

**Published:** 2025-02-28

**Authors:** Şeyda Doğantan, Sema Nur Taşkın, Cansu Yılmaz Yeğit, Ali Özdemir

**Affiliations:** 1 Department of Pediatric Rheumatology, Başakşehir Çam and Sakura City Hospital, 34480 İstanbul, Türkiye; 2 Department of Pediatric Rheumatology, Eskişehir City Hospital, 26080 Eskişehir, Türkiye; sntistanbul@gmail.com; 3 Department of Pediatric Chest Diseases, Başakşehir Çam and Sakura City Hospital, 34480 İstanbul, Türkiye; cansuuuuyilmaz@gmail.com; 4 Department of Pediatric Chest Diseases, Mersin City Hospital, 33330 Mersin, Türkiye; aliozdemir@hotmail.com

**Keywords:** juvenile idiopathic arthritis, pulmonary function tests, spirometry, lung diseases, interstitial, obstructive

## Abstract

Background/Objectives: Juvenile idiopathic arthritis (JIA) is a chronic autoimmune disorder characterized by joint inflammation, potentially leading to pulmonary involvement. This study aimed to assess pulmonary function in children with JIA compared to controls and identify potential respiratory abnormalities associated with the disease. Methods: This was a prospective cross-sectional study conducted at the Pediatric Rheumatology, Başakşehir Çam and Sakura City Hospital, İstanbul, Türkiye, between July and October 2024. The study included 70 children with JIA and 60 healthy controls aged 6 to 17. Pulmonary function test parameters, such as forced vital capacity (FVC), forced expiratory volume in the 1st second (FEV1), peak expiratory flow (PEF), and FEV1/FVC ratio, were measured using spirometry. Oxygen saturation (SpO_2_) was also measured. Results: There were no significant differences in demographic and clinical characteristics between the JIA and control groups (*p* > 0.05). FVC and FEV1 values were lower in the JIA group, though not significantly (*p* = 0.831 and *p* = 0.711). However, PEF was significantly lower in the JIA group than controls (*p* = 0.005). Children with moderate or high disease activity had significantly lower FVC, FEV1, and FEF 25–75 than those with low disease activity (*p* < 0.001). Enthesitis-related arthritis patients had higher FVC and FEV1 than other JIA subtypes (*p* < 0.05). FVC and FEV1 were positively correlated with BMI (*p* < 0.001). Conclusions: Although PEF values were significantly lower in children with JIA, overall pulmonary function was comparable between the groups. Regular pulmonary monitoring in JIA patients is recommended for early detection and management of respiratory complications.

## 1. Introduction

Juvenile idiopathic arthritis (JIA) is a chronic autoimmune and autoinflammatory musculoskeletal disorder that typically occurs before the age of 16. The exact etiology of JIA remains unclear. It can be confounding due to its common clinical and pathological features with adult rheumatoid arthritis [1]. However, pulmonary manifestations, including restrictive pulmonary patterns, decreased lung diffusion for carbon monoxide, and interstitial lung disease that is common in adult rheumatoid arthritis, are infrequent in pediatric JIA cases [1,2]. The prevalence of pulmonary involvement in JIA is estimated to be 4% to 8% [2]. JIA’s most common respiratory abnormalities are pneumonitis and pleuritis, characterized by transient or persistent pulmonary infiltrates [3,4].

Systemic JIA represents a distinct subtype characterized by joint inflammation, fever, rash, generalized lymphadenopathy, and, in some cases, hepatomegaly/splenomegaly and/or serositis [1,5,6,7,8]. Although pulmonary complications are rare, their occurrence, particularly in systemic JIA, poses significant morbidity and mortality risks, including pulmonary hypertension, interstitial lung disease, and pulmonary alveolar proteinosis [5,6,7].

Pulmonary function tests (PFTs) using spirometry are widely recognized as effective, cost-efficient, and readily available non-invasive tools for evaluating pulmonary impairment. In the context of rheumatic diseases and their potential respiratory involvement, PFTs might provide an objective means to assess pulmonary function, particularly restrictive ventilatory impairment, without the need for more invasive techniques such as plethysmography, the helium dilution method, nitrogen washout, or computed tomography [9,10]. Spirometry, in particular, is sufficiently sensitive for detecting early small airway involvement in various diseases [11], making it a valuable tool for identifying subclinical pulmonary dysfunction in JIA patients. While the diffusing capacity of the lung for carbon monoxide is a more sensitive early marker for pulmonary impairment, its limited availability restricts its widespread use [10]. Given the potential for subclinical pulmonary involvement in JIA and the need for accessible, routine monitoring, PFTs are a practical and clinically relevant approach to bridging the existing knowledge gap in respiratory assessment in these patients.

There are a limited number of studies in the literature investigating pulmonary function abnormalities indicating parenchymal involvement in children with JIA. In addition, it is controversial whether pulmonary abnormalities are due to pulmonary involvement of rheumatological disease or to the adverse effects of immunosuppressive therapy [1,2,3].

In this context, this study assessed pulmonary function in children with JIA and identified the demographic and clinical risk factors associated with impaired lung function.

## 2. Materials and Methods

This study was designed as a prospective cross-sectional study. The study protocol was approved by the Başakşehir Çam and Sakura City Hospital Clinical Research Ethics Committee (approval number: 2024-KAEK-11, approval date: 26 June 2024). The study was conducted by the ethical principles outlined in the Declaration of Helsinki. Written informed consent was obtained from the legal guardians of the children included in the study.

The population of this prospective cross-sectional study consisted of consecutive newly diagnosed JIA patients aged between 6 and 17 years followed up at the Outpatient Clinics of the Pediatric Rheumatology Department, Faculty of Medicine, Başakşehir Çam and Sakura City Hospital, İstanbul, Türkiye, between July and October 2024. JIA was diagnosed according to the International League of Associations for Rheumatology classification of JIA [12]. Among the newly diagnosed cases of JIA, those who were followed for at least 6 months were included in the study [2]. Children under 5 or over 18 years old and those with previous diagnoses of JIA, chronic lung diseases, or clinical evidence of pulmonary manifestations were excluded from the study. In the end, 70 children with JIA constituted the study sample (Group JIA), and 60 children who presented with complaints of extremity pain to the same outpatient clinics and whose age, gender, weight, and height characteristics matched the sample constituted the control group.

Post hoc power analysis was performed using G*Power 3.1.9.6 software to evaluate the statistical power of our study design. Due to the complex nature of pulmonary manifestations in JIA and the uncertainty in expected effect sizes from the previous literature, we conducted a comprehensive power analysis after data collection. Based on the primary outcome measure (PEF values) with an observed effect size (Cohen’s d) of 0.51, our final sample sizes of 70 JIA patients and 60 controls yielded 82.7% statistical power at an alpha level of 0.05, demonstrating adequate power to detect clinically meaningful differences between groups.

Demographic characteristics (age, gender) and weight and height measurements of the children included in the study were obtained from medical records and patient charts. Additionally, we prospectively collected clinical data notably regarding disease-related characteristics (passive exposure to smoking, physical activity grades, disease duration, grades of disease activity, categories of JIA, and treatment details). The physical activity of children with JIA was graded from sedentary to severe [13]. The disease severity of JIA was graded according to the Juvenile Arthritis Disease Activity Score (JADAS10) as mild-, moderate-, and severe disease activity classes [14]. The JADAS10 scores ranging from 1.1 to 2 for oligoarticular involvement and 1.1 to 3.8 for polyarticular involvement were used to describe the low-disease activity (LDA). The moderate disease-activity (MDA) group consisted of children with JADAS10 scores ranging from 2.1 to 4.2 for oligoarticular involvement and 3.9 to 10.5 for polyarticular involvement. The high-disease-activity (HDA) group encompassed children with JADAS10 scores exceeding 4.2 for oligoarticular involvement and 10.5 for polyarticular involvement. The International League of Associations for Rheumatology classification of JIA was used to categorize JIA cases [12].

Children’s oxygen saturation levels (SpO_2_, %) were measured before the pulmonary testing. An experienced technician performed the pulmonary function tests according to international recommendations using a Sensor Medics Corporation Spirometer (Model CA92687, SN 54065, Osaka, Japan) [15]. Within the scope of the pulmonary function tests, predicted forced vital capacity (FVC), predicted forced expiratory volume in the 1st second (FEV1), peak expiratory flow (PEF), and forced mid-expiratory flow (FEF 25–75) were measured. The results of the measurements were reported in percentages predicted based on equations [16,17]. The FEV1/FVC ratio was calculated. Each child was tested three times consecutively, and the best spirometric measurements were used in the analyses.

High-resolution computed tomography (HRCT) was performed in a subset of patients with suspected abnormal pulmonary function tests to evaluate potential interstitial lung disease, pleural thickening, bronchial wall thickening, ground-glass opacities, and other parenchymal abnormalities. An experienced pediatric radiologist, blinded to both clinical and spirometric data, reviewed all imaging findings.

All statistical analyses were performed using Jamovi (version 2.3.28) and JASP (version 0.18.3) software. The normality of continuous variables was assessed using the Shapiro–Wilk test (*n* < 50) and Kolmogorov–Smirnov and Anderson–Darling tests (*n* ≥ 50, *p* > 0.05), supported by visual inspection of histograms and Q-Q plots.

For group comparisons, Independent Samples T-test was used for normally distributed variables, while Mann–Whitney U test was applied for non-normal distributions. Categorical variables were analyzed using Pearson’s Chi-Square test. For multiple group comparisons (JIA subtypes), Kruskal–Wallis H test was performed, followed by Dwass-Steel–Critchlow–Fligner test for pairwise comparisons. Due to the non-normal distribution of variables, Spearman’s correlation coefficient was used to assess relationships between pulmonary function parameters and BMI.

Specific pulmonary function test parameters, including FVC, FEV1, and their percentage values, FEV1/FVC ratio, and oxygen saturation (SpO_2_), were compared between groups to assess potential differences. A *p*-value of ≤0.05 was considered statistically significant for all comparisons.

## 3. Results

There was no significant difference in demographic and clinical characteristics between the JIA and control groups (Table 1).

Table 2 presents the clinical characteristics of children with JIA regarding disease-related parameters. The median JADAS10 score was 3 among children with JIA. Based on the JADAS10 scoring system, most children (77.1%) had LDA, followed by MDA in 12 (17.1%) and HDA in four (5.7%). The most common JIA subtype was enthesitis-associated arthritis (42.9%), followed by oligoarthritis (25.7%) and polyarticular JIA (24.3%). The other characteristics are given in Table 2.

An analysis of the pulmonary function test results revealed that FVC and FEV1 were slightly lower, albeit not significantly, in the JIA group than in the control group (*p* = 0.831 and *p* = 0.711, respectively). In parallel, the groups had no significant difference in FEV1/FVC (*p* = 0.920). On the other hand, PEF was significantly lower in the JIA group than in the control group (78.5% vs. 89.0%, *p* = 0.005). There was no significant difference between the groups in other pulmonary function test parameters and oxygen saturation levels (*p* > 0.05) (Table 3).

Twenty-three children with JIA underwent HRCT scanning as part of their clinical evaluation following pulmonary function testing. All HRCT findings were interpreted as normal.

Although the percentage values of FEV1/FVC, PEF, and FEF 25–75 were similar in patients with LDA and those with MDA and HAD (*p* > 0.05), children with higher disease activity grades (MDA and HDA) had significantly lower percentage measurements of FVC, FEV1, and FEF 25–75 than those with LDA (*p* < 0.001 for all) (Table 4).

Comparison of pulmonary function tests in three main subcategories of JIA revealed significant differences (*p* < 0.05) (Table 4). Children with enthesitis-related arthritis had significantly higher FVC and FEV1 than those with oligoarthritis (*p* < 0.001 and *p* = 0.006, respectively) and polyarticular JIA (*p* = 0.001 and *p* = 0.011, respectively). SpO_2_ values were significantly lower in the enthesitis-related arthritis subcategory than in the oligoarthritis subcategory (*p* = 0.017). The other measurements were comparable between the groups (*p* > 0.05) (Table 5).

Significant correlations existed between some pulmonary function test parameters and BMI values for children with JIA (Table 5). FVC (r = 0.552, *p* < 0.001) and FEV1 (r = 0.634, *p* < 0.001) values were positively correlated with BMI values. Nevertheless, FEV1/FVC, PEF, and FEF 25–75 values did not correlate with the BMI values (*p* > 0.05) (Table 6).

## 4. Discussion

The study findings revealed that the pulmonary functions of children with JIA were comparable to those of the control subjects. As for the values that differed between the two groups, the PEF value was significantly lower, and the FVC and FEV1 values were slightly lower in the JIA group than in the control group. Significantly lower PEF values in the JIA group may be attributed to mild respiratory involvement secondary to JIA. Nevertheless, these findings highlighted a significant association between pulmonary function and disease activity in children with JIA. Children with higher disease activity exhibited significantly lower FVC, FEV1, and FEF 25–75 values than those with less severe disease. These results suggest that higher disease activity might be associated with a restrictive pulmonary pattern, potentially reflecting subclinical pulmonary involvement or the systemic inflammatory burden of JIA. So, these findings show the need for careful respiration monitoring in JIA patients, which is compatible with the literature regarding the pulmonary effects of JIA.

Data in the literature corroborate our findings that pediatric patients with JIA may exhibit subclinical pulmonary abnormalities, underlining the necessity for early pulmonary function testing. Early diagnosis and treatment prevent more severe pulmonary complications [18]. Screening and interventions for lung complications are critical in children with rheumatic diseases [19]. Nevertheless, the results of the pulmonary function tests of children with JIA reported in different studies are contradictory. In one of these studies, Alkady et al. [3] reported significant decreases in pulmonary function test parameters, including FVC, PEF, and carbon monoxide diffusion capacity of the lung, in children with JIA. They concluded that both restrictive and obstructive lung impairments are common in pediatric JIA cases. Significantly lower values of FVC and FEV1 have been reported in Indian children with JIA, reflecting the restrictive patterns of alterations in pulmonary function [1]. In a study on the changes in the diffusion capacity of the lungs in pediatric JIA cases, Attanasi et al. [2] found that the diffusion capacity significantly decreased in children with JIA compared with healthy controls. In contrast, other pulmonary function test parameters, including FEV1, FVC, FEF25–75, PEF, total lung capacity, and residual volume, did not significantly change.

Our study demonstrated that children with higher JIA disease activity exhibited significantly lower FVC, FEV1, and FEF 25–75 values, suggesting a restrictive pulmonary pattern, which aligns with previous reports indicating impaired lung function in JIA [1,2,3]. These discrepancies across studies, such as restrictive, obstructive, or combined pulmonary impairments, may be attributed to variations in disease duration, JIA subtypes, treatment regimens, and ethnic differences among study populations. Additionally, differences in the methodologies used to assess pulmonary function, including variations in reference values and testing conditions, may contribute to the inconsistencies in reported findings.

Subgroup analysis based on JIA categories revealed distinct differences in pulmonary function. Children with enthesitis-related arthritis had significantly higher FVC and FEV1 values than those with oligoarthritis and polyarticular JIA, indicating better lung function in this subgroup. However, the significantly lower SpO_2_ levels in enthesitis-related arthritis compared to oligoarthritis suggest possible alterations in oxygenation, which may warrant further investigation, clarifying the potential differences in pulmonary involvement across JIA subgroups.

Additionally, the positive correlation of FVC and FEV1 with BMI suggests that a higher body mass may be associated with better lung function parameters, potentially due to a greater lung volume or muscle mass. However, the absence of a significant correlation between BMI and FEV1/FVC, PEF, or FEF 25–75 suggests that factors beyond body composition may influence airway dynamics. These findings highlighted the importance of monitoring pulmonary function in children with JIA, particularly those with higher disease activity, to detect and manage potential respiratory complications early.

In comparison, in this study, we observed a significant difference between the JIA and control groups only in PEF. Previous studies have suggested that PEF is an objective measure of lung function, primarily associated with airway hyper-responsiveness. Additionally, it could predict asthma exacerbation and can be used in assessing the disease severity [20,21]. Mehta et al. [21] reported that PEF changes might detect airway obstruction, even in the pre-clinical asymptomatic stages. Similarly, the current study findings showed that the significant decrease in PEF in JIA patients might point to an aspect of respiratory involvement that may not be overtly symptomatic but may have long-term implications on pulmonary health. Although the association between PEF changes and asthma was the main speculative issue in these studies, the authors also speculated that anthropometric (age, height, and weight), sex, regional, and environmental parameters (altitude) might impact PEF measurements [22,23,24].

This finding is particularly intriguing as it raises questions about the subtle and possibly progressive respiratory involvement in JIA that may be overlooked in routine clinical assessments. The fact that we did not find any significant difference between the JIA and control groups in terms of other spirometric parameters suggests that primary pulmonary functions, such as mid-expiratory flow rates and oxygen saturation levels, are preserved despite the systemic nature of JIA. Thus, our findings indicate that although JIA can affect lung function, the resilience of the respiratory system remains significant overall.

It is stated in the literature that failure to perform pulmonary functional evaluation constitutes a reason for exclusion from the study. The most common problems that lead to impaired thoracic and/or spinal mobility and difficulty in performing all maneuvers for lung function tests in children with JIA are widespread muscular and/or skeletal disorders [3]. Such problems may be attributed to the severity of JIA or the medications patients use, including methotrexate [2,6,16,25,26,27]. In comparison, we did not observe extensive lung involvement that would prevent the completion of the pulmonary evaluation in any of our cases.

The literature also suggests alternative techniques, such as the interrupter technique, for assessing airway abnormalities in children with JIA [4]. However, given the limited reliability of spirometry in young children, we did not use such methods in our study.

The disproportionately high frequency of enthesitis-related arthritis in our study cohort compared to other JIA subtypes may be attributed to several demographic and methodological factors [3]. Notably, recent epidemiological data from Turkey indicate that enthesitis-related arthritis is the second most common JIA subtype after oligoarticular JIA, with a prevalence rate of 24.3%, which is higher than those reported in many Western populations [28]. Other studies from Turkey have also observed a high frequency of ERA, consistently identifying it as the second most common JIA subtype, with reported prevalence rates ranging from 18.9% to 23.2% [29,30,31]. This regional trend suggests that genetic and environmental factors unique to Turkey and neighboring regions may contribute to the observed distribution. Additionally, our study population had a median age of 14 years. In contrast, oligoarticular JIA predominantly affects younger children under six years of age, potentially leading to an underrepresentation of this subtype in our cohort.

Moreover, our study was conducted at a tertiary referral center specializing in pediatric rheumatology, where more complex and treatment-resistant cases, including those with enthesitis-related arthritis, are more likely to be referred. This referral bias contributed to the relatively high proportion of enthesitis-related arthritis cases. Furthermore, Turkey’s geographic position at the intersection of Europe and Asia, combined with increased migration from Asian regions where enthesitis-related arthritis is more prevalent, may have influenced the distribution of JIA subtypes in our study [3]. These factors highlight the importance of considering regional epidemiological variations and referral patterns when interpreting JIA subtype frequencies in different populations.

The primary limitation of this study was its cross-sectional design, which limited our ability to conclude the progression of pulmonary function over time in pediatric JIA patients. Longitudinal studies are needed to understand the dynamics of pulmonary changes that emerge as the disease progresses or treatment effects become apparent. Another limitation of the study was the sample size. At the same time, sufficient for initial comparisons, it was relatively small to detect subtle differences between groups regarding some pulmonary function test parameters or classify results into different JIA subtypes. Secondly, the sample size, although sufficient for initial comparisons, was relatively small to detect subtle differences between groups regarding some pulmonary function test parameters or to stratify the results by different JIA subtypes, which can be considered another study limitation. Future studies with larger cohorts are needed to delineate more specific patterns of pulmonary involvement across various JIA categories and confirm our findings’ generalizability. Thirdly, the possibility that standard pulmonary function tests may not detect early or mild forms of airway inflammation and remodeling that may be present in JIA patients may be an additional limitation of the study. Advanced imaging techniques, such as ultrasound, positron emission tomography, and the diffusing capacity of the lungs, or more sensitive biomarkers of the airway and parenchymal health, may provide a more comprehensive picture of the respiratory involvement in JIA [32,33]. Lastly, although we accounted for a range of confounding variables, additional, unmeasured factors, including environmental or genetic factors, the severity of JIA, and medications, may affect pulmonary outcomes in children with JIA. Addressing these factors in future studies may improve our understanding of the interplay between JIA and pulmonary health.

## 5. Conclusions

This study showed no significant differences in respiratory symptoms and spirometric parameters between pediatric JIA patients and control subjects. However, given the slight decreases in major pulmonary function parameters such as FVC and FEV1, we recommend a regular and periodic assessment of pulmonary function. Additionally, lower PEF values in JIA patients highlight a specific area of pulmonary involvement that may not be clinically evident but could affect the patient’s quality of life if not monitored and appropriately managed.

## Figures and Tables

**Table 1:** Demographic and clinical characteristics of children with JIA and healthy controls.

	Groups		
	Children with JIA (*n* = 70)	Healthy Controls (*n* = 60)	*p*-Values
Age (Years) ^§^	14.0 [6.0–17.0]	14.0 [6.0–17.0]	0.999 **
Gender ^‡^			
Female	44 (62.9)	37 (61.7)	0.999 ***
Male	26 (37.1)	23 (38.3)	
Height (cm) ^§^	158.0 [116.0–180.0]	158.0 [116.0–180.0]	0.828 **
Weight (kg) ^†^	49.0 [19.0–109.0]	49.0 [19.0–92.0]	0.885 **
Body mass index (kg/m^2^) ^†^	20.9 ± 4.7	20.7 ± 4.6	0.843 *

^‡^: *n* (%), ^†^: Mean ± standard deviation, ^§^: median [minimum–maximum]. JIA: Juvenile idiopathic arthritis. *. Independent Samples *t*-test. **. Mann–Whitney U test. ***. Pearson’s Chi-Square test.

**Table 2:** Clinical and disease characteristics of children with juvenile idiopathic arthritis.

Characteristics	Total (*n* = 70)
Passive smoke exposure ‡	36 (51.4)
Physical activity level ‡	
Sedentary	5 (7.1)
Mild	65 (92.9)
Disease characteristics	
Disease duration (months) §	3.0 [3.0–6.0]
JADAS10 score §	3.0 [3.0–37.0]
Disease activity ‡	
Low-disease activity	54 (77.1)
Moderate-disease activity	12 (17.1)
High-disease activity	4 (5.7)
JIA subtype ‡	
Enthesitis-related arthritis	30 (42.9)
Oligoarthritis	18 (25.7)
Polyarticular	17 (24.3)
Psoriatic arthritis	3 (4.3)
Systemic	2 (2.9)
Treatment history	
Previous medication ‡	
Methotrexate	70 (100.0)
NSAIDs	19 (27.1)
Systemic steroids	70 (100.0)
Current medication ‡	
Methotrexate only	44 (62.9)
Adalimumab	11 (15.7)
Tocilizumab	3 (4.3)
Etanercept	2 (2.9)

‡: *n* (%); § Data presented as median [minimum–maximum]. Abbreviations: JIA, juvenile idiopathic arthritis; JADAS10, Juvenile Arthritis Disease Activity Score based on 10 joints; Disease activity defined by JADAS10 criteria: Low (oligoarticular: 1.1–2.0; polyarticular: 1.1–3.8), Moderate (oligoarticular: 2.1–4.2; polyarticular: 3.9–10.5), High (oligoarticular: >4.2; polyarticular: >10.5).

**Table 3:** Pulmonary function tests and oxygen saturation in children with JIA and healthy controls.

	Groups		*p*-Values
	Children with JIA (*n* = 70)	Healthy Controls (*n* = 60)	
FVC (liters) ^†^	2.4 ± 0.9	-	-
FVC (%) ^†^	92.4 ± 10.1	92.8 ± 9.7	0.831 *
FEV1 (liters) ^†^	2.0 ± 0.8	-	-
FEV1 (%) ^†^	95.9 ± 10.9	96.6 ± 9.8	0.711 *
FEV1/FVC (%) ^§^	103.0 [81.0–114.0]	103.0 [81.0–114.0]	0.920 **
PEF (%) ^§^	78.5 [45.0–142.0]	89.0 [45.0–142.0]	0.005 **
FEF 25–75 (%) ^§^	99.5 [44.0–160.0]	98.0 [44.0–150.0]	0.452 **
SpO_2_ (%) ^§^	99.0 [94.0–100.0]	98.5 [94.0–100.0]	0.767 **

^†^: Mean ± standard deviation, ^§^: median [minimum–maximum]. JIA: juvenile idiopathic arthritis, FVC: predicted forced vital capacity, FEV1: predicted forced expiratory volume in the 1st second, PEF: peak expiratory flow, FEF25–75: forced mid-expiratory flow. *. independent samples *t*-test. **. Mann–Whitney U test. Statistically significant differences are indicated in bold.

**Table 4:** Comparison of pulmonary function tests and oxygen saturation in children with different disease activity grades.

	Disease Activity		*p* *
	LDA (*n* = 54)	MDA + HDA (*n* = 16)	
FVC (liters) ^§^	2.4 [0.9–4.2]	2.6 [0.6–4.2]	0.421
FVC (%) ^§^	96.5 [80.0–110.0]	84.0 [60.0–92.0]	**<0.001**
FEV1 (liters) ^§^	2.1 [0.9–3.6]	2.2 [0.3–3.6]	0.726
FEV1 (%) ^§^	100.5 [83.0–116.0]	82.5 [70.0–87.0]	**<0.001**
FEV1/FVC (%) ^§^	104.0 [89.0–113.0]	99.5 [81.0–114.0]	0.150
PEF (%) ^§^	81.0 [45.0–142.0]	72.0 [45.0–122.0]	0.081
FEF 25–75 (%) ^§^	101.5 [70.0–160.0]	82.0 [44.0–129.0]	**<0.001**
SpO_2_ (%) ^§^	98.5 [94.0–100.0]	99.0 [95.0–100.0]	0.785

^§^: Median [minimum–maximum]. LDA: low-disease activity, MDA: moderate-disease activity, HAD: high-disease activity, FVC: predicted forced vital capacity, FEV1: predicted forced expiratory volume in the 1st second, PEF: peak expiratory flow, FEF25–75: forced mid-expiratory flow. *. Mann–Whitney U test. Statistically significant differences are indicated in bold.

**Table 5:** Comparison of pulmonary function tests and oxygen saturation in children with different subcategories of JIA.

	Category of JIA			*p*-Values
	Oligoarthritis (*n* = 18)	Enthesitis-Related Arthritis (*n* = 30)	Polyarticular JIA (*n* = 17)	
FVC (liters) ^§^	1.8 [0.9–3.7]	2.8 [1.8–4.2]	1.8 [0.6–3.9]	**<0.001**
FVC (%) ^§^	96.0 [75.0–106.0]	91.0 [80.0–107.0]	92.0 [60.0–110.0]	0.930
FEV1 (liters) ^§^	1.7 [0.8–3.1]	2.4 [0.3–3.6]	1.7 [0.7–3.2]	**0.002**
FEV1 (%) ^§^	101.5 [82.0–115.0]	95.5 [77.0–114.0]	94.0 [70.0–115.0]	0.266
FEV1/FVC (%) ^§^	103.5 [97.0–109.0]	100.0 [83.0–113.0]	10 4.0 [81.0–114.0]	0.761
PEF (%) ^§^	89.0 [54.0–142.0]	77.0 [52.0–113.0]	72.0 [45.0–142.0]	0.065
FEF 25–75 (%) ^§^	101.5 [68.0–150.0]	88.0 [53.0–160.0]	88.0 [44.0–150.0]	0.163
SpO_2_ (%) ^§^	99.0 [95.0–100.0]	98.0 [94.0–100.0]	99.0 [96.0–100.0]	**0.011**

^§^: Median [minimum–maximum]. JIA: juvenile idiopathic arthritis, FVC: predicted forced vital capacity, FEV1: predicted forced expiratory volume in the 1st second, PEF: peak expiratory flow, FEF25–75: forced mid-expiratory flow. Statistically significant differences are indicated in bold.

**Table 6:** Correlation analysis of pulmonary function tests and oxygen saturation with body mass index values of children with JIA (*n* = 70).

	Body Mass Index	
	r	*p*
FVC (liters)	0.552	**<0.001**
FVC (%)	0.398	**<0.001**
FEV1 (liters)	0.634	**<0.001**
FEV1 (%)	0.264	**0.027**
FEV1/FVC (%)	−0.064	0.596
PEF (%)	−0.107	0.380
FEF 25–75 (%)	−0.101	0.405
SpO_2_ (%)	−0.387	**<0.001**

JIA: Juvenile idiopathic arthritis, FVC: predicted forced vital capacity, FEV1: predicted forced expiratory volume in the 1st second, PEF: peak expiratory flow, FEF25–75: forced mid-expiratory flow. Spearman’s rho correlation coefficient was used. Statistically significant differences are indicated in bold.

## Data Availability

The datasets used and analyzed during the current study are available from the corresponding author upon reasonable request. Restrictions may apply due to patient confidentiality and ethical guidelines.

## Abbreviations

The following abbreviations are used in this manuscript:

JIA	Juvenile Idiopathic Arthritis
PEF	Peak Expiratory Flow
FVC	Forced Vital Capacity
FEV1	Forced Expiratory Volume in the 1st Second
SpO_2_	Oxygen Saturation
